# Comparative investigation of different telemetric methods for measuring intracranial pressure: a prospective pilot study

**DOI:** 10.1186/s12987-020-00225-0

**Published:** 2020-10-17

**Authors:** S. Rot, M. Dweek, P. Gutowski, L. Goelz, U. Meier, J. Lemcke

**Affiliations:** 1 Department of Neurosurgery, Unfallkrankenhaus Berlin, Warener Straße 7, 12683 Berlin, Germany; 2 Department of Radiology and Neuroradiology, Unfallkrankenhaus Berlin, Warener Straße 7, 12683 Berlin, Germany

**Keywords:** NEUROVENT^®^-P-tel probe, Miethke sensor reservoir^®^, Intracranial pressure, External ventricle drainage

## Abstract

**Objectives:**

Measurement of intracranial pressure (ICP) plays an important role in long-term monitoring and neuro-intensive treatment of patients with a cerebral shunt. Currently, only two complete telemetric implants with different technical features are available worldwide. This prospective pilot study aims to examine patients who had both probes implanted at overlapping times for clinical reasons and represents the first in vivo comparison of both measurement methods.

**Materials and methods:**

Patients with a primary subarachnoid hemorrhage or a spontaneous intracerebral hemorrhage with ventricular hemorrhage who had received a telemetric ICP probe (Raumedic^®^ NEUROVENT^®^-P-tel) were included in the study. Conventional external ventricular drainages (EVD) and ventriculoperitoneal shunts with a telemetric ICP probe (Miethke Sensor Reservoir) were implanted in patients with hydrocephalus who required CSF (cerebrospinal fluid) drainage. Absolute ICP values from all systems were obtained. Due to the overlapping implantation time, parallel ICP measurements were performed via two devices simultaneously. ICP measurements via the sensor reservoir were repeated after 3 and 9 months. Differences between the absolute ICP values measured via the NEUROVENT^®^-P-tel probe, the Miethke sensor reservoir^®^, and the EVD were analyzed.

**Results:**

Seventeen patients were included in the present study between 2016 and 2018. 63% of all patients were male. In 11 patients the ICP measurements were followed up with both devices for 3 months. ICP measurements of the sensor reservoir showed corresponding trends in 9 cases compared to ICP measurement via the telemetry probe or EVD. Difference in absolute ICP values ranged between 14.5 mmHg and 0.0 mmHg. The average difference of the absolute ICP values in 8 cases was ≤ 3.5 mmHg.

**Conclusion:**

ICP measurements with both systems continuously showed synchronous absolute ICP values, however absolute values of ICP measurement with the different systems did not match.

## Background

A telemetric technique for intracranial pressure (ICP) measurements was first mentioned in 1965 by MacKey et al. [1, 2]. In the following years various telemetric ICP sensors were developed.

ICP monitoring in intubated patients is of great importance for the physicians while the patient cannot be assessed neurologically.

An acute or permanent increase in ICP as a result of direct or indirect damage to the brain parenchyma is associated with a poor neurological outcome [3].

In neuro-intensive care patients ICP monitoring is usually performed with an external ventricular drainage (EVD) or with a conventional wired ICP probe.

Since 2010, a telemetric probe, the NEUROVENT^®^-P-tel (Raumedic^®^, Helmbrechts, Germany) has been regularly used to measure ICP in intensive care patients [1, 2, 4, 5]. Once the NEUROVENT^®^-P-tel probe is implanted in the brain parenchyma via a borehole it is approved for a maximum implantation period of 3 months.

Telemetric ICP monitoring plays an important role in the examination of CSF dynamics in patients with suspected chronically elevated intracranial pressure.

Furthermore, the telemetric ICP probe NEUROVENT^®^-P-tel is becoming increasingly important in the diagnosis of suspected idiopathic normal pressure hydrocephalus [1, 2, 4–6]. As an example, ICP curves can be used to establish the indication for ventriculoperitoneal shunt placement.

Another application of telemetric ICP measurement is the maintenance of a shunt system. In order to provide the possibility to measure ICP within a ventriculoperitoneal (VP) shunt a fully implantable borehole reservoir with a measuring cell (Sensor Reservoir^®^, Miethke, Potsdam, Germany) was developed [7–9]. This device enables permanent non-invasive (transcutaneous) telemetric ICP measurements and thus the possibility of specific valve adjustments for individual patients.

In this study, we compared ICP values recorded via a telemetric probe and via a shunt-integrated sensor reservoir^®^ interindividually in patients with secondary hydrocephalus.

## Materials and methods

This study was approved by the ethics committee of the Charité–Universitätsmedizin Berlin and written consent was obtained from the patients or their legal guardians.

Patients between 18 and 80 years with primary subarachnoid (SAH) or spontaneous intracerebral hemorrhage (ICH) with ventricular hemorrhage were eligible for participation. Patients with traumatic brain injury and traumatic subarachnoid hemorrhage or secondary intracerebral hemorrhage on primary computed tomography (CT) were excluded from the study.

After emergency implantation of a NEUROVENT^®^-P-tel probe additional implantation of an external ventricular drainage ICP was monitored in the intensive care unit.

After initial implantation of the NEUROVENT^®^-P-tel probe and an EVD in eight patients, ICP was measured synchronously. ICP values were recorded and saved hourly during the first three days after implantation. Absolute ICP values of both measurements (EVD versus P-tel) for each hour were compared, see previous publication [10].

**Fig. 1:**
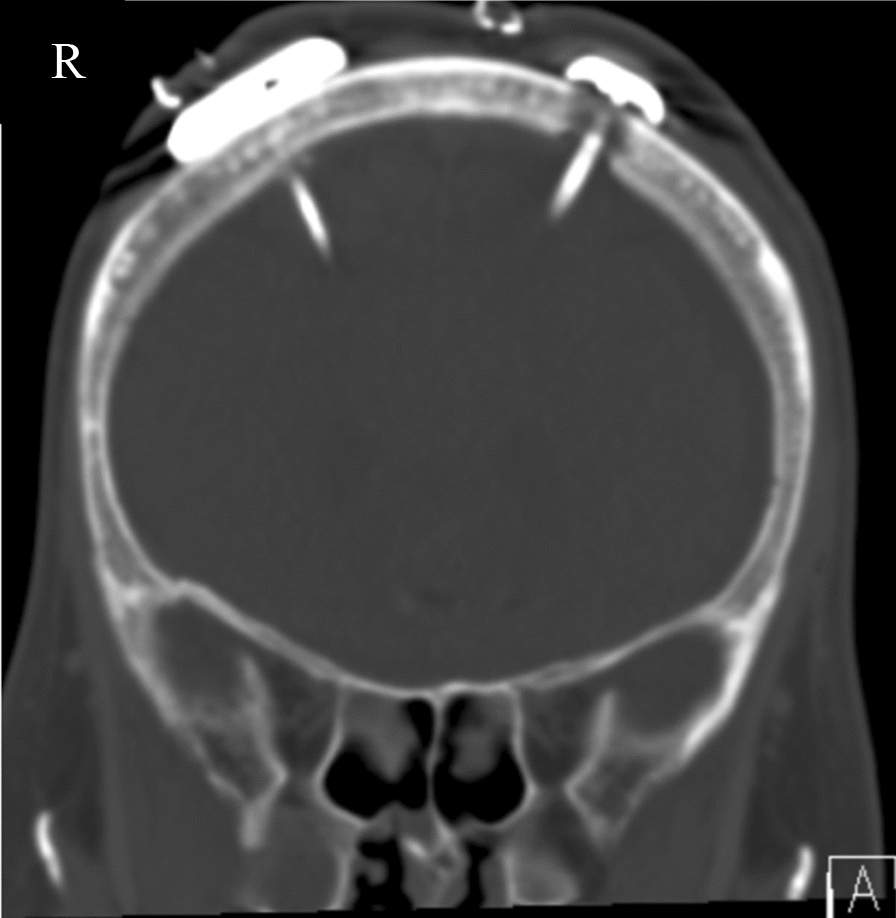
Coronar reformation of a cerebral CT after implantation of a NEUROVENT^®^-P-tel probe through a right frontal burr-hole and a sensor reservoir^®^ connected to a ventricular catheter through a left frontal burr-hole. (Institute for Radiology and Neuroradiology at the Unfallkrankenhaus Berlin, Director Prof. Dr. med. S. Mutze)

In patients who developed a secondary hydrocephalus (sNPH) a VP shunt (VPS) with sensor reservoir^®^ was implanted (Fig. 1). All patients with apparent potential for rehabilitation received adjustable pressure valves (proGAV, Aesculap-Miethke, Potsdam, Germany) with fixed gravitational valves (between 200 and 300 mm H_2_O according to body size).

In patients who would not be mobilized in the near future received adjustable valves without gravitational units set to 100 mmH_2_O initially.

After implantation of the VPS with sensor reservoir^®^, ICP was measured via both devices for the remaining implantation time of the NEUROVENT^®^-P-tel probe. During the first 10 days, ICP was measured three times per day (approximately every 8 h) for five minutes via sensor reservoir^®^ and NEUROVENT^®^-P-tel probe.

After three months the NEUROVENT^®^-P-tel probes were explanted. One day before explantation ICP was recorded via NEUROVENT^®^-P-tel probe and via sensor reservoir each for five minutes in lying (30° upper body high position), sitting or standing position of the patient. After explantation of the NEUROVENT^®^-P-tel probe, ICP was recorded via the sensor reservoir^®^ during follow-up examination after three months.

The readout in both devices works via radio-frequency identification (RFID) with specific antennas. The signal from the Raumedic^®^ read out device (TDT1 readP) for NEUROVENT^®^-P-tel probe generates a frequency of 5 Hz. The signal can be looped into an ICU monitor and processed with the integrated care management software (ICM, Dräger, Lübeck, Germany). The SENSOR RESERVOIR^®^ Reader Unit generates ICP values with a frequency of up to 40 Hz. The signal is stored on secure digital memory cards (SD cards) and cannot be looped in a medical monitor. The measuring cells at implantation sites were zeroed before each measurement. Three values per minute of ICP were recorded by each device (0, 30 and 60 s) and the mean value of ICP was calculated after five minutes for comparison.

Statistical evaluation was performed using Numbers for Mac OS and Microsoft Excel for Windows (Microsoft Corp.). The ICP values were analyzed using Pearsons correlation coefficient with IBM SPSS (version 25).

## Results

Between February 2016 and October 2018, 17 patients were included in this study. The mean age of the six female and ten male patients was 57 years (26–80). Eleven patients were included with spontaneous SAH and six patients with ICH with ventricular hemorrhage. One patient died in the intensive care unit (ICU) before and had to be excluded. 16 data sets were analyzed.

**Fig. 2:**
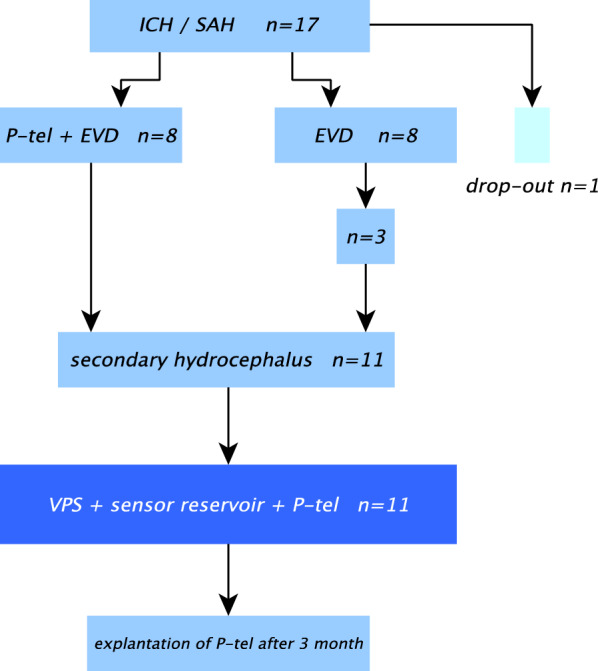
Flow diagram of the study protocol

In all patients an EVD was placed. In eight patients a NEUROVENT^®^-P-tel probe was implanted within the same session. Eleven of the 16 patients developed a secondary hydrocephalus and required VPS implantation: all patients of “P-tel + EVD group” (n = 8) and three of “EVD group” (Fig. 2). In these patients a VPS with sensor reservoir^®^ was implanted through a left frontal burr-hole while the NEUROVENT^®^-P-tel probe was implanted contralaterally in those that did not already have one (Fig. 2).

ICP measurements were initially performed in the ICU. ICP values were stored in the ICM software every hour. After implantation of the VPS, data was collected via NEUROVENT^®^-P-tel probe and the sensor reservoir.

We performed ICP measurements using the sensor reservoir^®^ and the NEUROVENT^®^-P-tel probe for three months before explantation of the NEUROVENT^®^-P-tel probe in 11 patients.

**Fig. 3:**
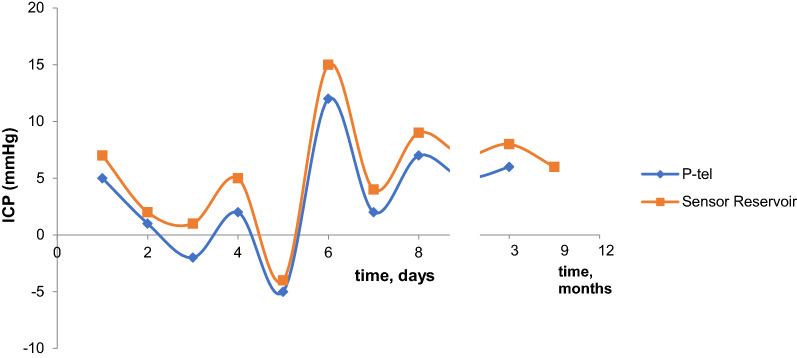
Parallel alignment of the absolute ICP values measured via the sensor reservoir^®^ and the NEUROVENT^®^-P-tel probe in a selected patient

The difference of the absolute ICP values between sensor reservoir^®^ and NEUROVENT^®^-P-tel probe showed a range of 0.0 and 14.5 mmHg. The mean value of the ICP difference was 4.2 mmHg with a standard deviation of 3.94 mmHg. The absolute ICP values from the sensor reservoir^®^ and the NEUROVENT^®^-P-tel probe presented parallel alignment in nine cases. As representative example of parallel alignment of absolute ICP values measured via sensor reservoir^®^ and the NEUROVENT^®^-P-tel probe in a selected patient is shown in Fig. 3. After three months the NEUROVENT^®^-P-tel probe was explanted and the measurement of ICP value via sensor reservoir^®^ was continued for the follow-up examination (Fig. 3).

**Fig. 4:**
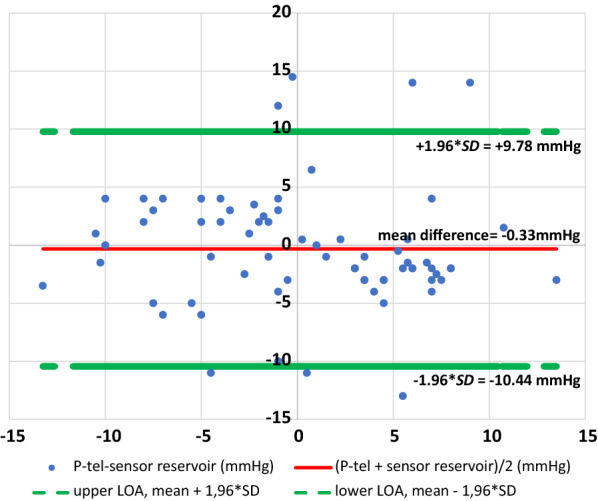
The Bland-Altman plot represents the agreement of both measuring methods (NEUROVENT^®^-P-tel probe and sensor reservoir^®^) in 11 patients. Blue dots: the difference of the ICP values; red line: mean difference; green lines: upper and lower limits of agreement (LOA)

The Bland–Altman plot of collected data of 11 patients after three months shows all measurements within 95% confidence interval. Thus, there is no systematic difference between the two measuring techniques (Fig. 4). The mean difference is – 0.33 mmHg.

The Pearson correlation was significant in nine cases (Table 1). ICP values were normally distributed (Shapiro–Wilk-Test: NEUROVENT^®^-P-tel: W = 0.98555, p = 0.61045; sensor reservoir^®^: W = 0.98528, p = 0.05210) [11]. Each of data point is an average of the measured absolute ICP values, which were determined by two measurement procedures on 1 day and in one patient. Table 1 shows that in patient no. 1 a total of six parallel measurements of NEUROVENT^®^-P-tel and sensor reservoir^®^ were performed in the period from day 1 after implantation of the sensor reservoir in secondary hydrocephalus until the last measurement before explantation of the NEUROVENT-P-tel (approved for max. 3 months) (see also Fig. 3). The absolute ICP values measured with the NEUROVENT^®^-P-tel probe were lower than the values measured with the sensor reservoir^®^ in six cases (55%). Figure 3 shows the representative curve of the two measuring methods.

**Table 1:** Difference of mean ICP values between NEUROVENT^®^-P-tel probe and sensor reservoir^®^

Case number	NDP of sensor reservoir^®^	NDP of NEUROVENT^®^-P-tel	Mean of ICP-difference	Correlation coefficient	p-values
1	6	6	2.66	0.877^a^	0.011
2	8	8	2.06	0.881^a^	0.002
3	7	7	3.92	0.934^a^	0.001
4	4	4	10.2	0.916^a^	0.014
5	6	6	2.75	0.657	0.078
6	10	10	2.1	0.991^a^	0.0
7	7	7	3.5	0.987^a^	0.0
8	4	4	13.6	0.979^a^	0.01
9	7	7	1.6	0.997^a^	0.0
10	6	6	2.8	0.856^a^	0.015
11	3	3	1.3	0.937	0.114

*NDP* number of data points, I*CP* intracranial pressure

^a^the significance level was defined as p ≤ 0.05

**Fig. 5:**
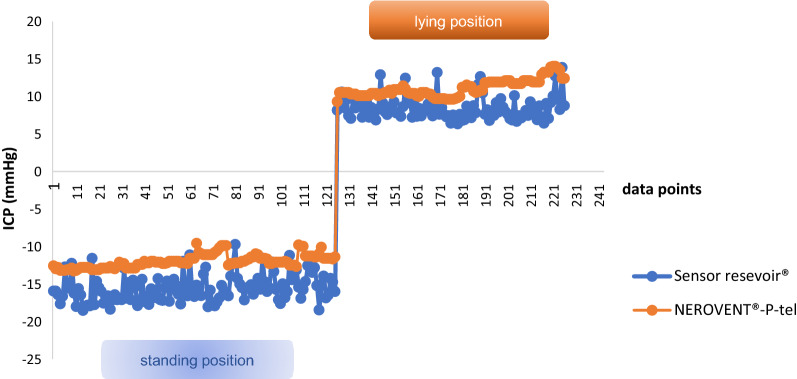
Dependency of ICP on the body position without implantation of a gravitational valve measured via sensor reservoir^®^ and NEUROVENT^®^-P-tel probe in comparison (an example in a selected patient)

A telemetric measurement with both the NEUROVENT^®^-P-tel probe and the sensor reservoir^®^ provided a comprehensible change in ICP values depending on the patient's body position (Fig. 5).

**Fig. 6:**
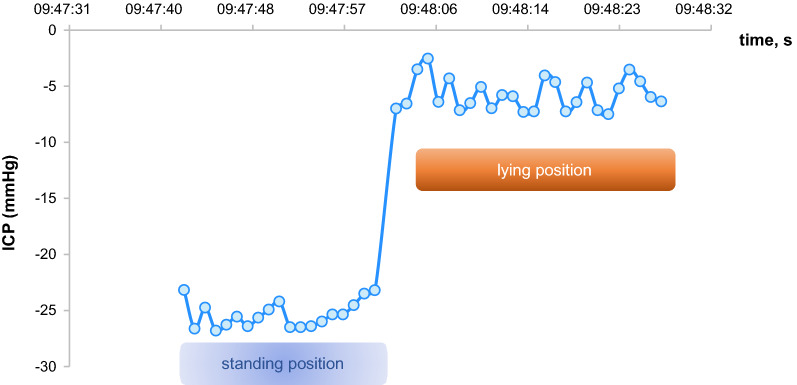
Dependency of ICP on the body position without implantation of a gravitational valve measured via sensor reservoir^®^ (an example in a selected patient)

**Fig. 7:**
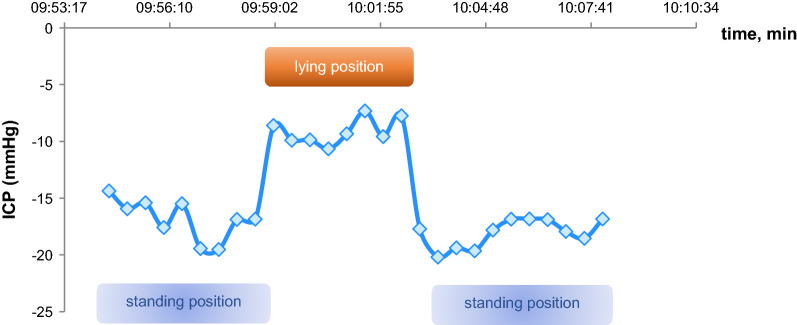
Dependency of ICP on the body position after implantation of a fixed gravitational valve measured via sensor reservoir^®^ (an example in a selected patient)

Figures 6, 7 show the ICP values measured via sensor reservoir after changing the patient's position. Figure 6 shows the ICP dynamics in VPS with a proGAV without gravitational valves. In Fig. 7 the ICP dynamics are shown in VPS with a proGAV with a fixed gravitational valve. The difference between ICP values in standing and lying position is significantly less in VPS with a fixed gravitational valve than without.

The measurement of ICP dynamics via NEUROVENT^®^-P-tel probe in relation to body position changes was successful in only three patients as they showed a good clinical outcome and were mobilizable. The ICP measurement via NEUROVENT^®^-P-tel probe shows similar dynamics of ICP change as the measurement via sensor reservoir^®^ in VPS with a proGAV without gravitational valves (Fig. 5).

In our current study, over-drainage occurred in only one patient approximately 3 months after implantation of a VP shunt with fixed differential pressure unit and integrated sensor reservoir^®^. The CT scan showed hygroma on both sides. The ICP measurement via the sensor reservoir was able to record the over-drainage and showed a value of − 17 mmHg in the standing position. The patient complained of headache symptoms.

## Discussion

Telemetric measurements of ICP becomes gain rising importance in modern neurosurgery. The NEUROVENT^®^-P-tel probe has been proven to be safe and effective in telemetric ICP monitoring and is used as reference in acute therapy of elevated ICP as well as for the diagnosis of neurological diseases such as pseudotumor cerebri and idiopathic normal pressure hydrocephalus (iNPH) [1, 2, 4, 5, 8, 12, 13].

The sensor reservoir^®^ (Miethke, Aesculap, Germany) is available to determine pressure conditions continuously within a shunt system and thus to assess over- or under-drainage during shunt therapy. Radiation exposure of CT scan or long and expensive MRI for the diagnosis of over- or under-drainage symptoms can thus be avoided.

Since the approval of the sensor reservoir^®^ for clinical use in 2015, no direct prospective comparison with another established system for measuring ICP has been available.

### Management of telemetric devices

All devices were implanted for clinical reasons according to their licensed purpose, following the routine procedures. During the time of parallel implantation absolute ICP values gained by NEUROVENT^®^-P-tel probes and VP shunts with sensor reservoir^®^ could be compared directly.

The advantage of both telemetric systems is the possibility of continued monitoring after transfer to the neurosurgical ward, rehabilitation, or domesticity, as well as during different body positions of the patient.

However, the absolute ICP values collected with both measuring devices were within the clinical normal range in each case, so that no contradictory therapeutic consequences resulted from the measured values of the different systems.

To the best of our knowledge there are three publications about the sensor reservoir^®^ [7, 8, 14].

Antes and colleagues [8] who co-developed the sensor reservoir^®^ implanted the sensor reservoir^®^ in patients who had already received conventional VPS and were suspected to have suboptimal valve settings. The sensor reservoir^®^ was thus used to try to detect shunt-associated complications such as over- and underdrainage in time and to treat them with appropriate changes in valve settings.

Ertl et al. [7] used the sensor reservoir^®^ to analyze the change in ICP values in relation to the change in the patient's body position. Our study confirms his findings in standing and lying position.

The most recent study is a technical note by Norager et al. [14], who describes technical advantages and disadvantages and present one illustrative case for each device.

A comparison of measured ICP values in the cerebral CSF compartment and the brain parenchyma has already been described in the literature [10, 15–18]. Brean, Eide et al. [10] analyzed the ICP dynamics of wired intraventricular and parenchymatous ICP probes in comparison. In some patients with primary SAH, a parenchymatous sensor was implanted alongside the EVD in the same hemisphere. The ICP values were then measured simultaneously using both devices. In contrast, a one-sided implantation of both measuring cells of telemetric devices proved to be impracticable in the present study due to the design of the corresponding reading devices. For this reason, both hemispheres were used for implantation in our patient population even though imbalanced intracranial pathologies could have tampered the results. However, the positive correlation of ICP curves for both devices indicates comparable measurements while differences of absolute ICP values could be attributed to implantation site, calibration, and other technical issues. This difference in measurement is to be expected and can be justified on the one hand by the different measurement situations of the two measurement sensors. The sensor reservoir^®^ measures the CSF pressure changes that occur on a corrugated, biocompatible membrane, which changes are passed on to the pressure sensor via a chamber filled with air or special gas [9]. In this case, the measurement location is the reservoir that is implanted in the calotte. The NEUROVENT^®^-P-tel probe is a piezo- resistive pressure sensor that is located on the tip of a 3 cm long intra-parenchymatous catheter. The pressure transducer contains several electrical resistors that are doped on a flexible membrane. This membrane is in direct contact with the pulsating brain tissue. An increase in the ICP leads to an expansion of the membrane. These changes in resistance are registered by a pressure transducer and converted into ICP values [2,19]. In this case, the measurement location is approximately 3 cm deep. The different absolute ICP values can presumably be attributed to a hydrostatic pressure difference [10].

Due to the elastic properties of the shunt catheter, it is assumed that the transmission of pulsating ICP components is damped [7]. In addition, the ICP measured via the sensor reservoir depends in part on the valve setting.

A technical error could also arise. The technical error rate of the sensor reservoir is 8% [8], and that of the intraparenchymal ICP probes is 3–16% [1, 2, 8, 20, 21]. Another factor that could explain the differences in the absolute values is the zero-point drift of both measurement methods. A zero drift of the NEUROVENT^®^-P-tel of  ± 2.5 mmHg has already been described in the literature [2, 5, 21].

### ICP measurement via sensor reservoir^®^ versus NEUROVENT^®^-P-tel probe

In this study, the absolute ICP values of the sensor reservoir^®^ did not match the absolute ICP values of the NEUROVENT^®^-P-tel probe. However, the difference of the mean ICP values was ± 4 mmHg (1.3–13.6 mmHg). This difference was to be expected and can be explained by the different measurement locations of both sensors, elastic properties of the shunt, by technical errors or by zero-point drift (see section above).

However, the tendency of the ICP dynamics of both systems is largely synchronous in the present study despite the difference between the absolute ICP values. The correlation coefficient was significant in nine cases (81.8%).

The study also shows that ICP values change accordingly to the patient’s position. In the case of the programmable differential valves without gravitational unit measured via sensor reservoir^®^ and NEUROVENT^®^-P-tel probe in the same patient, the pressure gradient between lying and standing position was significantly greater than in patients with additionally implanted fixed or adjustable gravitational unit. The regulation of the CSF outflow rate during standing position and thus the avoidance of over-drainage complications with the existing gravitational valve can be recorded with ICP continuously monitoring via the sensor reservoir^®^. This information can help during diagnosis and therapy of over-drainage. The telemetrically acquired ICP data can also be used to determine the indication for implantation of an additional gravitational unit. The SVASONA study showed the efficacy of gravitational units through avoidance of over-drainage complications for patients with idiopathic normal pressure hydrocephalus [22, 23]. Using the adjustable proSA valve (Miethke, Germanmy) as a gravitational unit, the shunt system can be adjusted even more precisely to the individual needs of a patient.

A disadvantage of the sensor reservoir is that the large RFID antenna of the reading device must be placed over the sensor reservoir in order to measure and store the ICP values. A permanent fixation of the heavy antenna on the head of patient is not possible with the current version of the device. A long-term ICP monitoring for 24–48 h to determine the Lundberg A and B waves is therefore not possible [8].

Because the sensor reservoir has a height of 7.7 mm, there is also a cosmetic disadvantage after implantation because a swelling remains visible. A wound dehiscence above the sensor reservoir was not observed in our hospital but seems possible in elderly patients or thinned skin.

In the study by Ertl et al. [7] the sensor reservoir was implanted in two patients with normal pressure hydrocephalus. A measurement with a frequency of 1 Hz was performed. As in our study, a similar change in ICP values was measured depending on changes in the patients' body position. It can thus be concluded that the sensor reservoir^®^ provides traceable real-time values. In our opinion this telemetric technique can be used for the diagnosis and therapy of over- or under-drainage in shunt patients.

Freimann et al. [12] implanted a NEUROVENT^®^-P-tel probe in addition to the programmable shunt valve in four patients with hydrocephalus. In these patients telemetric ICP measurements were helpful in valve adjustment and enabled regular evaluation of the position-dependent ICP values as a therapeutic target. However, the NEUROVENT^®^-P-tel probe can be implanted for only 3 months. The sensor reservoir^®^, on the other hand, enables permanent ICP measurement. In the work of Antes [8] the sensor reservoir^®^ was implanted in 25 patients. Complications such as over- or underdrainage could also be detected and quantified with the sensor reservoir^®^. The valves could be individually adjusted according to ICP measurements. This study was unique in describing the use of the sensor reservoir^®^ was to diagnose shunt complications. An additional adjustable gravitational unit (pro-SA) was implanted, which was connected distally of the fixed gravitational unit. During the control examination after 4 months ICP measurements showed a reduction in to – 7 mmHg and clinical improvement with reduced headaches was observed.

## Limitations

As a limitation of the study is the partially incomplete data collection should be mentioned. Parallel measurements of the ICP via the sensor reservoir^®^ and NEUROVENT^®^-P-tel probe for approx. 5 min 3 times a day was a great challenge despite the 24-h service in our clinic. Sometimes only one ICP value was obtained via both telemetric devices.

In addition, the small number of cases is a significantly limiting factor.

## Conclusion

Absolute ICP values of the sensor reservoir^®^ and the NEUROVENT^®^-P-tel probe do not absolutely concur during parallel measurements through bihemispheric burr-holes in patients with primary SAH or ICH with intraventricular hemorrhage but both devices show correlating ICP dynamics. During changing of body positions of patients the sensor reservoir^®^ demonstrate static differences in ICP dynamics.

The data provides a comparison measurement using the newer sensor reservoir^®^ and the already known NEUROVENT^®^-P-tel probe under daily life conditions. Based on these promising results, we believe that the use of the sensor reservoir can be an additional tool for the detection and treatment control of drainage-associated complications in shunt implantation.

## Data Availability

The datasets used during the current study are available from the corresponding author on reasonable request.

## Abbreviations

SAH: Subarachnoid hemorrhage
ICH: Intracerebral hemorrhage
sNPH: Secondary normal pressure hydrocephalus
VPS: Ventriculoperitoneal shunt
CSF: Cerebrospinal fluid
cCT: Cerebral computer tomography
P-tel: Parenchymatous-telemetric
EVD: External ventricular drainages
ICP: Intracranial pressure
ICU: Intensive care unit
ICM: Integrated care management software
iNPH: Idiopathic normal pressure hydrocephalus
